# Efficacy of Amphetamine Extended-Release Oral Suspension in Children with Attention-Deficit/Hyperactivity Disorder: Effect Size Across the Day

**DOI:** 10.1089/cap.2022.0093

**Published:** 2023-02-14

**Authors:** Stephen V. Faraone, Ann C. Childress, Roberto Gomeni, Eman Rafla, Judith C. Kando, Lori Dansie, Payal Naik, Antonio Pardo

**Affiliations:** ^1^ SUNY Upstate Medical University, Syracuse, New York, USA.; ^2^ Center for Psychiatry and Behavioral Medicine, Inc., Las Vegas, Nevada, USA.; ^3^ PharmacoMetrica, La Fouillade, France.; ^4^ Tris Pharma, Inc., Monmouth Junction, New Jersey, USA.

**Keywords:** amphetamine, attention-deficit/hyperactivity disorder, extended-release, laboratory school, oral suspension, liquid formulation, effect size

## Abstract

**Objective:**

To evaluate the treatment effect size throughout the day of amphetamine extended-release oral suspension (AMPH EROS; Tris Pharma, Inc., Monmouth Junction, NJ, USA) in a laboratory classroom study conducted in children aged 6–12 years with attention-deficit/hyperactivity disorder (ADHD).

**Methods:**

A *post hoc* analysis was performed to assess the overall effect size as well as the effect size at each time point from early morning through evening (1, 2, 4, 6, 8, 10, 12, and 13 hours postdose) for each efficacy measure evaluated in a 5-week, randomized, dose-optimized, double-blind, placebo-controlled, laboratory classroom assessment, efficacy, and safety study of AMPH EROS (*N* = 99). Change from baseline of the primary (Swanson, Kotkin, Agler, M-Flynn, Pelham [SKAMP]-C) and key secondary (secondary efficacy assessments included the SKAMP attention [SKAMP-A], SKAMP-deportment subscale [SKAMP-D], Permanent Product Measure of Performance-number of problems attempted [PERMP-A], PERMP-number of problems correct [PERMP-C]) efficacy measures were analyzed using a linear mixed model repeated-measures analysis model. Comparisons among treatments were adjusted for multiple comparisons using the Bonferroni method. The effect size was estimated using Cohen's *d*, to determine “small,” (0.2), “medium,” (0.5), or “large” (0.8) magnitudes of treatment effects.

**Results:**

Large overall effect sizes were observed for all primary and key secondary efficacy assessments. Moreover, the SKAMP-C, PERMP-number of problems attempted, and PERMP-C scores showed large effect sizes at each time point evaluated across the day, from 1 to 13 hours postdose. The SKAMP-A and SKAMP-D scores showed a medium to large effect size at each time point.

**Conclusions:**

AMPH EROS demonstrated a large and consistent effect size across the day, including early in the morning, in the treatment of symptoms of ADHD in children aged 6–12 years. Trial Registration: clinicaltrials.gov identifier: NCT02083783

## Introduction

Attention-deficit/hyperactivity disorder (ADHD) is recognized as a widespread neurodevelopmental disorder with major public health implications worldwide (Faraone et al, 2021, Faraone et al, 2015). Treatment with stimulants has been the mainstay of pharmacotherapy for ADHD since the late 1930s (US Department of Health and Human Services, 2000). There are a broad variety of methylphenidates and amphetamines currently available for use (Perugi et al, 2019) as well as several nonstimulant medications that clinicians can prescribe for their patients with ADHD. One method for assessing the efficacy and safety of stimulant medications for the treatment of ADHD uses a randomized double-blind placebo-controlled study design conducted in either an analog laboratory classroom setting, for children, or a simulated workplace environment, for adults.

These studies typically evaluate efficacy by comparing the active drug treatment with placebo using a range of ADHD rating scales (ADHD-RSs) including the ADHD-RS; the Swanson, Kotkin, Agler, M-Flynn, Pelham (SKAMP) complete and subscale scores (Wigal and Wigal, 2006; Wigal et al, 1998); the Adult ADHD Investigator Symptom Rating Scale (AISRS) (Spencer et al, 2010); and the Permanent Product Measure of Performance (PERMP) (Wigal and Wigal, 2006). Efficacy is evaluated by comparing outcome measures at various time points during the day. These studies give adequate clinical evidence for evaluating the efficacy and time course of effect of medication relative to placebo and have been employed for assessments of efficacy for regulatory approval purposes for decades.

As the number of available agents for treatment of ADHD increases, the need for evaluation of one agent relative to another becomes of increasing importance. Direct comparisons of efficacy data from studies, whether by direct comparisons of mean difference in change relative to placebo, or by comparing *p*-values is problematic for a number of reasons including the use of different endpoints and assessment schema, as well as differences in precision between studies (Faraone et al, 2003).

Therefore, an effect size estimate places an interpretable value on the direction and magnitude of an effect of a treatment. This measure of effect can then be used to compare the efficacy of the treatment in question with similarly computed measures of treatment efficacy in other studies that may use seemingly noncomparable measures (Faraone and Buitelaar, 2010). The standardized mean difference effect size of a treatment is defined as the difference in improvement between drug and placebo after adjusting for precision of measurement (Faraone, 2008).

Amphetamine extended-release oral suspension (AMPH EROS; Tris Pharma, Inc., Monmouth Junction, NJ, USA) is an amphetamine base product, 3.2:1 mixture of *d-* and *l*-amphetamine, and was approved by the U.S. Food and Drug Administration in 2015 for the treatment of ADHD in patients aged 6–17 years. The indication was later expanded to patients aged 6 years and older. AMPH EROS utilizes Tris Pharma's proprietary Liqui*XR*^®^ ion-exchange drug-delivery technology that allows rapid release of active drug followed by a sustained extended release. The release characteristics of the Liqui*XR* drug-delivery technology are programmable and allow for a customized sustained release of active drug product for up to 24 hours postdose.

The technology consists of ionically binding drug particles to a micron-sized resin, which is then covered with a permeable coating. Mechanistically, after ingestion drug particles leave the resin and enter the gastrointestinal (GI) tract. To accomplish this, positively charged ions from the GI fluids diffuse across the coating where they displace the ionically charged drug product, which diffuses back through the coating and into the GI fluids for absorption. As the coating is of variable thickness, some drug product takes longer to diffuse and absorb, providing for the programmable delayed drug release characteristic (Herman et al, 2021).

The efficacy and safety of AMPH EROS as a treatment for ADHD were previously established in a laboratory classroom study (Childress et al, 2018). In that study, 108 boys and girls aged 6–12 years diagnosed with ADHD were enrolled in a 5-week open-label dose optimization phase and titrated to optimal dose (or maximum dose of 20 mg/day) of AMPH EROS. During the subsequent double-blind phase, subjects were randomized to receive either their optimal dose (10–20 mg/day) of AMPH EROS or placebo for 1 week. Efficacy was assessed in a laboratory classroom setting on the final day of double-blind treatment using the Swanson, Kotkin, Agler, M-Flynn, and Pelham-Combined (SKAMP-C) Rating Scale and PERMP math test.

The primary efficacy endpoint was change from predose SKAMP-C score at 4 hours postdose. Secondary endpoints were the change from predose SKAMP-C scores at intervals from 1 to 13 hours postdose. Onset and duration of efficacy were defined as the first and last measured time point that reached statistical significance. The study was completed by 99 subjects. The primary efficacy endpoint was met (least-squares [LS] mean treatment difference [95% confidence interval; CI] of −14.8 [−17.9 to −11.6], *p* < 0.0001). For key secondary efficacy endpoints, the onset of treatment effect occurred at the earliest time point assessed, 1-hour postdose (treatment difference LS mean [SE], −10.2 [1.61], *p* < 0.0001).

The duration of efficacy persisted until the final time point at 13 hours postdose (treatment difference LS mean [SE], −9.2 [1.61], *p* < 0.0001). At each postdose time point measured throughout the laboratory classroom day, the improvement from postdose SKAMP-C score was statistically significant (*p* < 0.0001) from 1 to 13 hours postdose. PERMP change scores from predose were also statistically significantly improved from 1 to 13 hours postdose.

### Study objectives

The objective of this *post hoc* analysis was to facilitate evaluation of the therapeutic effect of AMPH EROS in the treatment of children with ADHD as measured in a laboratory school environment. We calculated the overall effect size for this dose-optimized population of children aged 6–12 years with ADHD. We also examined the differences in magnitude of effect sizes for the SKAMP-C and associated subscales and the PERMP throughout the day from 1 through 13 hours postdose. Effect size data can provide practical evaluation of treatment effect of AMPH EROS versus other available agents and help guide treatment choices.

## Methods

**FIG. 1.**
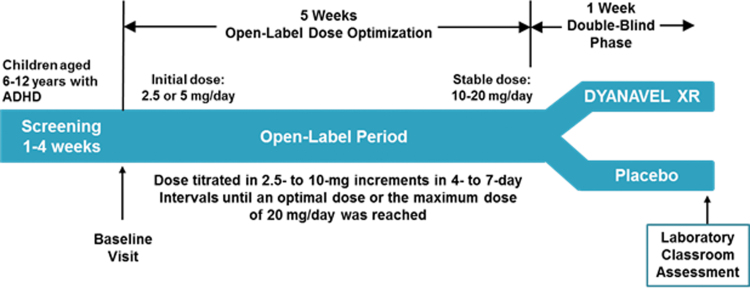
Study design. Adapted from figure 1 in Childress et al (2018).

In the initial Phase 3 study, boys and girls aged 6–12 years diagnosed with ADHD were enrolled. The overall study design is provided in Figure 1, and the complete details of the inclusion and exclusion criteria, methods, and conduct of the phase 3 study have been reported elsewhere (Childress et al, 2018).

### Efficacy measures

Efficacy assessments were performed on the intent-to-treat (ITT) population, defined as all randomized subjects who received at least one dose of double-blind study medication and had at least one postdose assessment of the primary efficacy variable (SKAMP-C). The SKAMP-C (Wigal and Wigal, 2006; Wigal et al, 1998) is a 13-item 7-point impairment scale that evaluates manifestations of ADHD in a classroom setting and includes two derivative subscales, attention and deportment (Wigal and Wigal, 2006; Wigal et al, 1998).

The PERMP is a 5-page timed written test that measures the number of math problems attempted and solved correctly in 10 minutes (Wigal and Wigal, 2006). The SKAMP-C is utilized in laboratory classroom settings because it is a direct observation scale, as opposed to the Swanson, Nolan, and Pelham (Swanson et al, 1983) or the ADHD-RS, which are rated by parents and caregivers, or by investigators based on an interview with adult caregivers. The SKAMP-C includes symptoms and behaviors that are characteristic of ADHD and also disruptive behavior disorders more broadly. Efficacy data were analyzed according to the treatment to which the subject was randomized.

Efficacy assessments were collected at predose, 1, 2, 4, 6, 8, 10, 12, and 13 hours postdose. The primary efficacy outcome was the onset of efficacy for AMPH EROS compared with placebo as assessed by the primary outcome measure, SKAMP-C scores (Childress et al, 2018). Key secondary efficacy assessments included the SKAMP attention (SKAMP-A) subscale and the SKAMP-deportment subscale (SKAMP-D); and the PERMP-number of problems attempted (PERMP-A) and PERMP-number of problems correct (PERMP-C). Different versions of the PERMP (with differing degrees of difficulty) were administered to subjects based on individual ability as assessed by a math pretest completed by each subject at the baseline visit.

### Effect size calculation

The efficacy variables used in this analysis were the change from baseline of the following scores: SKAMP-C, SKAMP-A, SKAMP-D, PERMP-A, and PERMP-C. These variables were analyzed using a linear mixed model repeated-measures analysis. The model included terms for treatment, time, predose, treatment-by-time interaction, predose-by-time interaction, study center, subject age, and subject sex. The random effects were specified using the repeated statement to account for serial within-subject correlation. A significance level *α* = 0.05 was deployed to establish the significance of treatment effect, which was determined using the mixed-effect model adjusted means (least-squares means [LS MEANS]). The LS MEANS methodology computed the LS MEANS of fixed effects. The comparisons among treatment accounted for the multiple comparison adjustment using the Bonferroni method.

The effect size was estimated for each variable and for each measurement using Cohen's *d* method, as the differences between drug effects and placebo effects (treatment effect) divided by their pooled standard deviation. Based on Cohen, effect sizes of 0.2, 0.5, and 0.8 were considered to correspond to a “small,” “medium,” and “large” magnitude of treatment effect (Cohen, 1992). Directionally speaking, negative results with SKAMP scores and positive scores with PERMP scores are indicative of improvement. All subjects with one dose of double-blind study medication and at least one postdose assessment of the selected variables were included in the analysis without imputation of missing data. The statistical analyses were conducted using the PROC MIXED procedure in SAS (Version 9.4 for Windows; SAS Institute, Cary, NC, USA).

## Results

Study enrollment included 108 participants, with 107 included in the enrolled safety population and 100 of these in the randomized safety population. A total of 99 participants (AMPH EROS, *n* = 51; placebo, *n* = 48) completed the study and were included in the ITT population. Nine participants discontinued from the study, eight during the open-label phase and one after randomization to AMPH EROS. In the open-label phase, the primary reason (4/8 patients; 50.0%) for study discontinuation was termination of a study site by the sponsor due to lack of participant enrollment; after randomization, one participant discontinued because of an illness on a laboratory school study day. More boys (68.7%) than girls participated in the study. The study population was 55.6% White and most participants had inattentive or combined type ADHD presentations. Complete participant characteristics are outlined (Table 1).

**Table 1.** Participant Characteristics

Subjects	Placebo (*n* = 48)	AMPH EROS (*n* = 51)	Total (*N* = 99)
Sex			
Male	32 (66.7)	36 (70.6)	68 (68.7)
Female	16 (33.3)	15 (29.4)	31 (31.3)
Age, years			
Mean	9.6 (1.76)	9.2 (1.95)	9.4 (1.86)
Median	10.0	9.0	9.0
Range (min, max)	(6, 12)	(6, 12)	(6, 12)
Race			
White	28 (58.3)	27 (52.9)	55 (55.6)
Black/African American	15 (31.3)	19 (37.3)	34 (34.3)
Other	5 (10.4)	5 (9.8)	10 (10.1)
Ethnicity			
Hispanic/Latino	21 (43.8)	18 (35.3)	39 (39.4)
Non-Hispanic/Latino	27 (56.3)	33 (64.7)	60 (60.6)
ADHD type			
Predominantly inattentive	8 (16.7)	12 (23.5)	20 (20.2)
Predominantly hyperactive/impulsive	1 (2.1)	0	1 (1.0)
Combined	39 (81.3)	39 (76.5)	78 (78.8)

Values are shown as *n* (%) unless otherwise noted. Percentages based on the number of subjects in each group.

ADHD, attention-deficit/hyperactivity disorder; AMPH EROS, amphetamine extended-release oral suspension.

### Efficacy analyses

#### SKAMP-combined

**FIG. 2.**
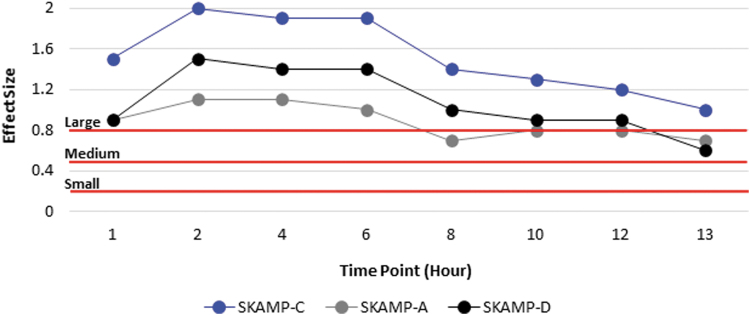
SKAMP effect size analysis. SKAMP-C, Swanson, Kotkin, Agler, M-Flynn, and Pelham Scale (SKAMP)-Complete Score; SKAMP-A, Swanson, Kotkin, Agler, M-Flynn, and Pelham Scale-attention subscale score; SKAMP-D, Swanson, Kotkin, Agler, M-Flynn, and Pelham Scale-deportment subscale score.

**FIG. 3.**
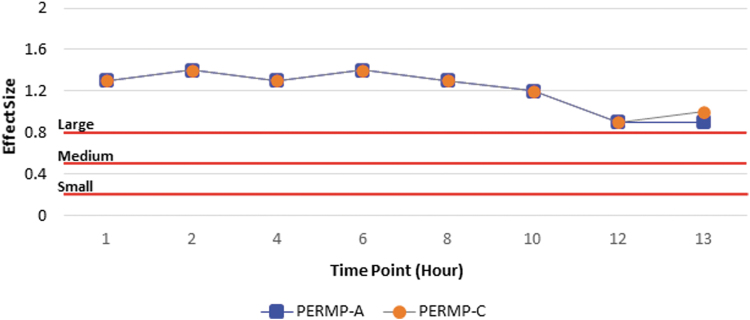
PERMP effect size analysis. PERMP-A, Permanent Product Measure of Performance-Problems attempted; PERMP-C, Permanent Product Measure of Performance-Problems correct.

All standardized mean difference effect sizes comparing AMPH EROS with placebo are listed by time point (Table 2). A total of 51 subjects from the AMPH EROS arm and 48 subjects from the placebo arm were included in the effect size calculations. The overall SKAMP-C effect size was 1.8, with large effect sizes noted at all time points assessed, from 1 to 13 hours postdose. The overall effect size for AMPH EROS on SKAMP-A was 1.1, with large effect sizes corresponding to all time points assessed (Fig. 2). The overall effect size for AMPH EROS on SKAMP-D was 1.3, with large effect sizes noted across all corresponding time points. The overall effect size for AMPH EROS on PERMP-A was 1.5, with large effect sizes consistently noted from hour 1 through 13. The overall effect size of AMPH EROS on PERMP-C was 1.1, with correspondingly large effect sizes noted at each individual point evaluated (Fig. 3).

**Table 2.** Effect Size Analysis

Time point (hour)	SKAMP-C	SKAMP-A	SKAMP-D	PERMP-A	PERMP-C
1	1.5^*^	0.9^*^	0.9^*^	1.3^*^	1.3^*^
2	2^*^	1.1^*^	1.5^*^	1.4^*^	1.4^*^
4	1.9^*^	1.1^*^	1.4^*^	1.3^*^	1.3^*^
6	1.9^*^	1^*^	1.4^*^	1.4^*^	1.4^*^
8	1.4^*^	0.7^*^	1^*^	1.3^*^	1.3^*^
10	1.3^*^	0.8^*^	0.9^*^	1.2^*^	1.2^*^
12	1.2^*^	0.8^*^	0.9^*^	0.9^*^	0.9^*^
13	1^*^	0.7^*^	0.6^*^	0.9^*^	1^*^
Overall	1.8^*^	1.1^*^	1.3^*^	1.5^*^	1.1^*^

^*^ *p*-Value <0.05.

PERMP, Permanent Product Measure of Performance; PERMP-A, PERMP-number of problems attempted; PERMP-C, PERMP-number of problems correct; SKAMP, Swanson, Kotkin, Agler, M-Flynn, Pelham; SKAMP-A, secondary efficacy assessments included the SKAMP attention; SKAMP-C, Swanson, Kotkin, Agler, M-Flynn, and Pelham-Combined; SKAMP-D, SKAMP-deportment subscale.

## Discussion

Clinical studies utilize *p*-values, a measure of statistical significance, to demonstrate that a reported finding is not likely due to chance (Faraone, 2008; McGough and Faraone, 2009). Although useful, *p*-values alone do not provide a complete picture of the efficacy of a treatment. In this *post hoc* analysis, the standardized mean difference effect size was used to incorporate significance, direction, magnitude, and relevance to provide a clinically meaningful picture of the observed effects. A large overall effect size was observed for all primary and key secondary efficacy assessments at each time point. Moreover, the SKAMP-C, PERMP-A, and PERMP-C scores showed a large effect size at each time point from 1 to 13 hours postdose; the SKAMP-A and SKAMP-D scores showed a medium to large effect size at each time point.

The onset of time of clinical effect was defined as the earliest time point at which the difference between placebo and AMPH EROS response was statistically significant. The onset of response was the same for all clinical scores assessed. The larger effect size was noted for SKAMP-C indicating that this clinical score was most sensitive to treatment with AMPH EROS. All scores reached statistical significance within 1 hour postdose, a result confirmed by an exploratory analysis of time to onset of AMPH EROS, which showed onset at 30 minutes postdose in a population of 18 subjects (Childress et al, 2018). In the present assessment there was a significant treatment effect for the PERMP-C at the earliest time point measured, which was 1 hour postdose.

Meta-analyses to measure the effect size for efficacy of stimulants based on performance measurements in the patient's environment, have found amphetamines, as a class, to range from medium to large effects (0.85–1.19) for the treatment of ADHD in children and adolescents (Cortese et al, 2018). The results presented here demonstrate medium to large effect sizes (0.6–1.8) for AMPH EROS in the treatment of children and adolescents with ADHD based on behavior measurements from a laboratory classroom environment. Although these findings are not directly comparable with effects from the patient's environment, they do show that AMPH EROS has a large peak effect that begins early in the morning, remains consistent throughout the core of the day, and slowly tapers down in the evening hours.

This study should be evaluated in the context of some limitations. First, as these are *post hoc* analyses and not part of the *a priori* planned analyses, they were not adequately powered to make direct comparisons of subgroup differences in efficacy variables. A second limitation is the relatively small sample size, which gives a less precise estimate of the effect size and less generalizability.

In summary, ADHD symptoms can affect aspects of life outside of school and work, such as socializing, driving, doing homework, and functioning in the family environment. Therefore, there is a need for medication that can treat patients throughout the day (Faraone et al, 2015). This *post hoc* analysis shows robust effect sizes for the SKAMP and PERMP at each time point from 1 to 13 hours postdose, which indicates that participants had clinical efficacy throughout the day and into the evening.

## Conclusion

AMPH EROS demonstrated robust and consistent effects beginning early in the morning and continuing throughout the day in the treatment of symptoms of ADHD in children aged 6–12 years.

## Clinical Significance

ADHD symptoms can affect aspects of life outside of school and work, such as socializing, driving, doing homework, and functioning in the family environment. Therefore, there is a need for medication that can treat patients throughout the day. An ideal psychopharmacological treatment of ADHD symptoms includes a rapid onset of effect postdose, with a duration of effect that provides efficacy through the morning and afternoon and into the early evening hours. The data provided in this article, combined with earlier efficacy and safety data, suggest that AMPH EROS shows consistent effect sizes at each time point from 1 to 13 hours postdose, indicating clinical efficacy throughout the day and into the evening.
